# 4-Benzyl-1-*p*-tolyl-1*H*-1,2,4-triazol-5(4*H*)-one

**DOI:** 10.1107/S1600536809002487

**Published:** 2009-01-23

**Authors:** Yu-Jin Zhu, Hong-Quan Duan

**Affiliations:** a School of Pharmacy, Tianjin Medical University, Tianjin 300070, People’s Republic of China; b School of Chinese Armed Police Force Medical College, Tianjin 300162, People’s Republic of China

## Abstract

In the title compound, C_16_H_15_N_3_O, the triazole ring makes dihedral angles of 7.08 (2) and 74.53 (3)° with the two outer aromatic rings. The crystal packing is stabilized by very short inter­molecular C—H⋯O hydrogen bonds and weak π–π stacking inter­actions [centroid-to-centroid distance 3.632 (3) Å], resulting in the formation of zigzag chains parallel to the *b* axis.

## Related literature

For details of the biological activity of tris­ubstituted triazol­inones, see: Chang *et al.* (1993 ▶, 1994 ▶). For bond-length data, see: Allen *et al.* (1987 ▶). For details of synthesis, see: Theodoridis (1998 ▶).
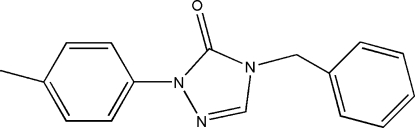


## Experimental

### 

#### Crystal data

C_16_H_15_N_3_O
*M* *_r_* = 265.31
Monoclinic, 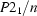

*a* = 4.6130 (9) Å
*b* = 25.488 (5) Å
*c* = 11.460 (2) Å
β = 96.18 (3)°
*V* = 1339.6 (5) Å^3^
*Z* = 4
Mo *K*α radiation
μ = 0.09 mm^−1^
*T* = 113 (2) K
0.18 × 0.04 × 0.04 mm

#### Data collection

Rigaku Saturn diffractometer
Absorption correction: multi-scan (*CrystalClear*; Rigaku, 2005 ▶) *T*_min_ = 0.985, *T*_max_ = 0.997
9828 measured reflections
2333 independent reflections
1998 reflections with *I* > 2σ(*I*)
*R*_int_ = 0.052

#### Refinement

*R*[*F* ^2^ > 2σ(*F* ^2^)] = 0.055
*wR*(*F* ^2^) = 0.150
*S* = 1.10
2333 reflections
183 parameters
H-atom parameters constrained
Δρ_max_ = 0.23 e Å^−3^
Δρ_min_ = −0.26 e Å^−3^

### 

Data collection: *CrystalClear* (Rigaku, 2005 ▶); cell refinement: *CrystalClear*; data reduction: *CrystalClear*; program(s) used to solve structure: *SHELXTL* (Sheldrick, 2008 ▶); program(s) used to refine structure: *SHELXTL*; molecular graphics: *SHELXTL*; software used to prepare material for publication: *SHELXTL*.

## Supplementary Material

Crystal structure: contains datablocks I, global. DOI: 10.1107/S1600536809002487/hg2470sup1.cif

Structure factors: contains datablocks I. DOI: 10.1107/S1600536809002487/hg2470Isup2.hkl

Additional supplementary materials:  crystallographic information; 3D view; checkCIF report

## Figures and Tables

**Table 1:** Hydrogen-bond geometry (Å, °)

*D*—H⋯*A*	*D*—H	H⋯*A*	*D*⋯*A*	*D*—H⋯*A*
C9—H9⋯O1^i^	0.93	2.19	3.114 (2)	174

Symmetry code: (i) 
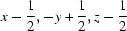
.

**Fig. 1.**
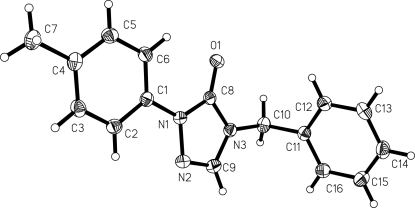
View of the title compound (I), with displacement ellipsoids drawn at the 40% probability level.

## supplementary crystallographic information

### Comment

4-Benzyl-1-*p*-tolyl-1*H*-1,2,4-triazol-5(4*H*)-one is a N-substituted triazolinone. It was reported that trisubstituted triazolinones were employed as nonpeptide angiotensin II receptor antagonists (Chang *et al.*, 1993, 1994). In our effort to further study triazolinone derivatives as novel AII antagonists, the title compound was prepared. Here, we report the crystal structure of it.

In title compound, all bond lengths in the molecule are normal (Allen *et al.*, 1987). The triazole ring N1–N3/C8–C9 makes dihedral angles of 7.08 (2) and 74.53 (3)° with the two phenyl rings (C1–C6, C11–C16). The relatively short distance of 3.632 (3) Å between the centroids of triazole ring N1–N3/C8–C9 and benzene ring C1–C6 [at -1 + *x*, *y*, *z*] indicates the presence of weak π–π interactions, The crystal packing is stabilized by intermolecular C—H···O hydrogen bonds, linking the molecules into zigzag chains parallel to the *b* axis.

### Experimental

1-*p*-Tolyl-1*H*-1,2,4-triazol-5(4*H*)one (1.75 g, 0.01 mol) was dissolved in 30 ml of acetic anhydride, 1.38 g (0.01 mol) potassium carbonate and 0.75 ml (0.01 mol) phenylmethanol were added. The solution was heated to reflux and stirred for 2 h and then cooled to room temperature. 100 ml of water was added and the deposited precipitate filtered. The precipitate was recrystallized with aetone and dried to give 4-benzyl-1-*p*-tolyl-1*H*-1,2,4-triazol-5(4*H*)one as a colorless power (2.40 g, yield 90.5%) (Theodoridis, 1998). Crystals suitable for X-ray diffraction were obtained through slow evaporation of the solution of the title compound in dichloromethane and ethyl acetate (v/v 1:1).

### Refinement

All H atoms were placed in calculated positions, with C—H = 0.93–0.97 Å,, and included in the final cycles of refinement using a riding model, with *U*_iso_(H) = 1.2 (1.5 times for methyl) times *U*_eq_(C).

### Crystal data

**Table:**

C_16_H_15_N_3_O	*F*(000) = 560
*M**_r_* = 265.31	*D*_x_ = 1.315 Mg m^−^^3^
Monoclinic, *P*2_1_/*n*	Mo *K*α radiation, λ = 0.71073 Å
Hall symbol: -P 2yn	Cell parameters from 2910 reflections
*a* = 4.6130 (9) Å	θ = 1.6–27.9°
*b* = 25.488 (5) Å	µ = 0.09 mm^−^^1^
*c* = 11.460 (2) Å	*T* = 113 K
β = 96.18 (3)°	Block, colourless
*V* = 1339.6 (5) Å^3^	0.18 × 0.04 × 0.04 mm
*Z* = 4	

### Data collection

**Table:**

Rigaku Saturn diffractometer	2333 independent reflections
Radiation source: rotating anode	1998 reflections with *I* > 2σ(*I*)
confocal	*R*_int_ = 0.052
ω scans	θ_max_ = 25.0°, θ_min_ = 2.0°
Absorption correction: multi-scan (*CrystalClear*; Rigaku, 2005)	*h* = −5→5
*T*_min_ = 0.985, *T*_max_ = 0.997	*k* = −30→30
9828 measured reflections	*l* = −13→13

### Refinement

**Table:**

Refinement on *F*^2^	Secondary atom site location: difference Fourier map
Least-squares matrix: full	Hydrogen site location: inferred from neighbouring sites
*R*[*F*^2^ > 2σ(*F*^2^)] = 0.055	H-atom parameters constrained
*wR*(*F*^2^) = 0.150	*w* = 1/[σ^2^(*F*_o_^2^) + (0.0922*P*)^2^] where *P* = (*F*_o_^2^ + 2*F*_c_^2^)/3
*S* = 1.10	(Δ/σ)_max_ < 0.001
2333 reflections	Δρ_max_ = 0.23 e Å^−^^3^
183 parameters	Δρ_min_ = −0.26 e Å^−^^3^
0 restraints	Extinction correction: *SHELXTL* (Sheldrick, 2008), Fc^*^=kFc[1+0.001xFc^2^λ^3^/sin(2θ)]^-1/4^
Primary atom site location: structure-invariant direct methods	Extinction coefficient: 0.030 (5)

### Special details

**Table:**

Geometry. All e.s.d.'s (except the e.s.d. in the dihedral angle between two l.s. planes) are estimated using the full covariance matrix. The cell e.s.d.'s are taken into account individually in the estimation of e.s.d.'s in distances, angles and torsion angles; correlations between e.s.d.'s in cell parameters are only used when they are defined by crystal symmetry. An approximate (isotropic) treatment of cell e.s.d.'s is used for estimating e.s.d.'s involving l.s. planes.
Refinement. Refinement of *F*^2^ against ALL reflections. The weighted *R*-factor *wR* and goodness of fit *S* are based on *F*^2^, conventional *R*-factors *R* are based on *F*, with *F* set to zero for negative *F*^2^. The threshold expression of *F*^2^ > σ(*F*^2^) is used only for calculating *R*-factors(gt) *etc*. and is not relevant to the choice of reflections for refinement. *R*-factors based on *F*^2^ are statistically about twice as large as those based on *F*, and *R*- factors based on ALL data will be even larger.

### Fractional atomic coordinates and isotropic or equivalent isotropic displacement parameters (Å^2^)

**Table:**

	*x*	*y*	*z*	*U*_iso_*/*U*_eq_	
O1	0.4812 (3)	0.20822 (5)	0.27648 (11)	0.0298 (4)	
N1	0.5904 (3)	0.17809 (5)	0.09066 (12)	0.0250 (4)	
N2	0.5011 (4)	0.19124 (6)	−0.02578 (13)	0.0328 (4)	
N3	0.2711 (3)	0.24033 (5)	0.09506 (13)	0.0267 (4)	
C1	0.7986 (4)	0.13699 (6)	0.11575 (15)	0.0247 (4)	
C2	0.8840 (4)	0.10765 (7)	0.02333 (16)	0.0295 (5)	
H2	0.8061	0.1147	−0.0533	0.035*	
C3	1.0867 (4)	0.06763 (7)	0.04622 (16)	0.0310 (5)	
H3	1.1441	0.0483	−0.0162	0.037*	
C4	1.2065 (4)	0.05553 (7)	0.15959 (17)	0.0281 (5)	
C5	1.1164 (4)	0.08567 (7)	0.25051 (17)	0.0305 (5)	
H5	1.1928	0.0784	0.3272	0.037*	
C6	0.9155 (4)	0.12638 (7)	0.23019 (16)	0.0284 (5)	
H6	0.8605	0.1462	0.2923	0.034*	
C7	1.4263 (4)	0.01155 (7)	0.18258 (18)	0.0337 (5)	
H7A	1.6175	0.0243	0.1720	0.051*	
H7B	1.3764	−0.0167	0.1287	0.051*	
H7C	1.4243	−0.0009	0.2616	0.051*	
C8	0.4527 (4)	0.20823 (6)	0.16844 (16)	0.0242 (4)	
C9	0.3125 (4)	0.22863 (7)	−0.01808 (17)	0.0328 (5)	
H9	0.2161	0.2456	−0.0828	0.039*	
C10	0.0864 (4)	0.28203 (7)	0.13604 (18)	0.0306 (5)	
H10A	0.0539	0.2752	0.2169	0.037*	
H10B	−0.1016	0.2817	0.0891	0.037*	
C11	0.2243 (4)	0.33566 (7)	0.12782 (15)	0.0265 (5)	
C12	0.4397 (4)	0.35251 (7)	0.21405 (16)	0.0326 (5)	
H12	0.4966	0.3310	0.2779	0.039*	
C13	0.5705 (5)	0.40130 (8)	0.20553 (18)	0.0370 (5)	
H13	0.7158	0.4121	0.2631	0.044*	
C14	0.4840 (5)	0.43383 (7)	0.11114 (17)	0.0374 (5)	
H14	0.5691	0.4667	0.1057	0.045*	
C15	0.2713 (5)	0.41719 (8)	0.02540 (18)	0.0388 (5)	
H15	0.2142	0.4389	−0.0381	0.047*	
C16	0.1420 (4)	0.36866 (7)	0.03274 (17)	0.0328 (5)	
H16	−0.0006	0.3579	−0.0259	0.039*	

### Atomic displacement parameters (Å^2^)

**Table:**

	*U*^11^	*U*^22^	*U*^33^	*U*^12^	*U*^13^	*U*^23^
O1	0.0334 (8)	0.0304 (7)	0.0250 (7)	0.0016 (5)	0.0005 (6)	−0.0033 (5)
N1	0.0277 (9)	0.0245 (8)	0.0224 (8)	0.0005 (6)	0.0004 (6)	0.0006 (6)
N2	0.0443 (10)	0.0300 (9)	0.0235 (9)	0.0029 (8)	0.0013 (7)	0.0019 (7)
N3	0.0273 (9)	0.0239 (8)	0.0284 (9)	0.0020 (6)	0.0012 (7)	0.0001 (6)
C1	0.0235 (10)	0.0226 (9)	0.0282 (10)	−0.0032 (7)	0.0032 (8)	0.0008 (7)
C2	0.0331 (11)	0.0297 (10)	0.0266 (10)	−0.0023 (8)	0.0066 (8)	0.0018 (7)
C3	0.0328 (11)	0.0295 (10)	0.0326 (11)	−0.0003 (8)	0.0125 (9)	−0.0039 (8)
C4	0.0217 (10)	0.0250 (9)	0.0384 (11)	−0.0046 (7)	0.0063 (8)	0.0005 (8)
C5	0.0273 (11)	0.0337 (11)	0.0295 (10)	−0.0012 (8)	−0.0012 (8)	0.0006 (8)
C6	0.0288 (11)	0.0274 (10)	0.0285 (11)	0.0000 (8)	0.0013 (8)	−0.0038 (7)
C7	0.0286 (11)	0.0288 (10)	0.0440 (12)	0.0015 (8)	0.0053 (9)	0.0009 (9)
C8	0.0244 (10)	0.0217 (9)	0.0258 (10)	−0.0045 (7)	0.0002 (7)	−0.0009 (7)
C9	0.0377 (12)	0.0308 (10)	0.0287 (11)	0.0021 (8)	−0.0019 (9)	0.0032 (8)
C10	0.0258 (11)	0.0271 (10)	0.0395 (11)	0.0012 (8)	0.0056 (9)	0.0002 (8)
C11	0.0265 (10)	0.0251 (9)	0.0292 (10)	0.0033 (7)	0.0088 (8)	−0.0008 (7)
C12	0.0383 (12)	0.0303 (10)	0.0292 (10)	0.0032 (8)	0.0035 (9)	−0.0023 (8)
C13	0.0376 (12)	0.0362 (11)	0.0365 (12)	−0.0032 (9)	0.0013 (9)	−0.0107 (9)
C14	0.0443 (13)	0.0280 (10)	0.0421 (13)	−0.0082 (9)	0.0151 (10)	−0.0037 (9)
C15	0.0486 (14)	0.0325 (11)	0.0360 (12)	−0.0005 (9)	0.0075 (10)	0.0066 (9)
C16	0.0332 (12)	0.0321 (10)	0.0319 (11)	0.0009 (8)	−0.0008 (9)	0.0002 (8)

### Geometric parameters (Å, °)

**Table:**

O1—C8	1.231 (2)	C7—H7A	0.9600
N1—C8	1.382 (2)	C7—H7B	0.9600
N1—N2	1.394 (2)	C7—H7C	0.9600
N1—C1	1.429 (2)	C9—H9	0.9300
N2—C9	1.299 (2)	C10—C11	1.515 (2)
N3—C9	1.364 (2)	C10—H10A	0.9700
N3—C8	1.388 (2)	C10—H10B	0.9700
N3—C10	1.471 (2)	C11—C12	1.392 (3)
C1—C2	1.388 (2)	C11—C16	1.397 (3)
C1—C6	1.390 (2)	C12—C13	1.390 (3)
C2—C3	1.390 (3)	C12—H12	0.9300
C2—H2	0.9300	C13—C14	1.387 (3)
C3—C4	1.391 (3)	C13—H13	0.9300
C3—H3	0.9300	C14—C15	1.379 (3)
C4—C5	1.394 (3)	C14—H14	0.9300
C4—C7	1.516 (2)	C15—C16	1.379 (3)
C5—C6	1.394 (3)	C15—H15	0.9300
C5—H5	0.9300	C16—H16	0.9300
C6—H6	0.9300		
			
C8—N1—N2	112.01 (14)	O1—C8—N1	129.94 (16)
C8—N1—C1	128.55 (15)	O1—C8—N3	126.98 (16)
N2—N1—C1	119.43 (14)	N1—C8—N3	103.07 (15)
C9—N2—N1	104.01 (15)	N2—C9—N3	112.84 (16)
C9—N3—C8	108.06 (15)	N2—C9—H9	123.6
C9—N3—C10	127.37 (16)	N3—C9—H9	123.6
C8—N3—C10	124.41 (15)	N3—C10—C11	111.73 (14)
C2—C1—C6	120.14 (16)	N3—C10—H10A	109.3
C2—C1—N1	118.77 (16)	C11—C10—H10A	109.3
C6—C1—N1	121.10 (15)	N3—C10—H10B	109.3
C1—C2—C3	119.47 (17)	C11—C10—H10B	109.3
C1—C2—H2	120.3	H10A—C10—H10B	107.9
C3—C2—H2	120.3	C12—C11—C16	118.79 (17)
C2—C3—C4	122.05 (17)	C12—C11—C10	120.45 (16)
C2—C3—H3	119.0	C16—C11—C10	120.75 (17)
C4—C3—H3	119.0	C13—C12—C11	120.55 (18)
C3—C4—C5	117.13 (17)	C13—C12—H12	119.7
C3—C4—C7	121.17 (17)	C11—C12—H12	119.7
C5—C4—C7	121.71 (17)	C14—C13—C12	119.93 (19)
C4—C5—C6	122.13 (18)	C14—C13—H13	120.0
C4—C5—H5	118.9	C12—C13—H13	120.0
C6—C5—H5	118.9	C15—C14—C13	119.65 (18)
C1—C6—C5	119.08 (17)	C15—C14—H14	120.2
C1—C6—H6	120.5	C13—C14—H14	120.2
C5—C6—H6	120.5	C14—C15—C16	120.78 (19)
C4—C7—H7A	109.5	C14—C15—H15	119.6
C4—C7—H7B	109.5	C16—C15—H15	119.6
H7A—C7—H7B	109.5	C15—C16—C11	120.30 (18)
C4—C7—H7C	109.5	C15—C16—H16	119.8
H7A—C7—H7C	109.5	C11—C16—H16	119.8
H7B—C7—H7C	109.5		
			
C8—N1—N2—C9	−0.07 (19)	C9—N3—C8—O1	178.18 (18)
C1—N1—N2—C9	179.13 (15)	C10—N3—C8—O1	2.4 (3)
C8—N1—C1—C2	172.54 (16)	C9—N3—C8—N1	−0.95 (18)
N2—N1—C1—C2	−6.5 (2)	C10—N3—C8—N1	−176.71 (14)
C8—N1—C1—C6	−7.7 (3)	N1—N2—C9—N3	−0.6 (2)
N2—N1—C1—C6	173.26 (15)	C8—N3—C9—N2	1.0 (2)
C6—C1—C2—C3	0.1 (3)	C10—N3—C9—N2	176.61 (16)
N1—C1—C2—C3	179.85 (15)	C9—N3—C10—C11	−75.1 (2)
C1—C2—C3—C4	0.5 (3)	C8—N3—C10—C11	99.78 (19)
C2—C3—C4—C5	−0.5 (3)	N3—C10—C11—C12	−80.5 (2)
C2—C3—C4—C7	179.72 (15)	N3—C10—C11—C16	98.3 (2)
C3—C4—C5—C6	−0.1 (3)	C16—C11—C12—C13	−0.1 (3)
C7—C4—C5—C6	179.67 (16)	C10—C11—C12—C13	178.78 (17)
C2—C1—C6—C5	−0.7 (3)	C11—C12—C13—C14	0.7 (3)
N1—C1—C6—C5	179.57 (15)	C12—C13—C14—C15	−0.9 (3)
C4—C5—C6—C1	0.7 (3)	C13—C14—C15—C16	0.4 (3)
N2—N1—C8—O1	−178.45 (17)	C14—C15—C16—C11	0.3 (3)
C1—N1—C8—O1	2.4 (3)	C12—C11—C16—C15	−0.4 (3)
N2—N1—C8—N3	0.64 (18)	C10—C11—C16—C15	−179.28 (17)
C1—N1—C8—N3	−178.46 (15)		

### Hydrogen-bond geometry (Å, °)

**Table:**

*D*—H···*A*	*D*—H	H···*A*	*D*···*A*	*D*—H···*A*
C9—H9···O1^i^	0.93	2.19	3.114 (2)	174

Symmetry codes: (i) *x*−1/2, −*y*+1/2, *z*−1/2.
